# Mechanisms involved in breast cancer liver metastasis

**DOI:** 10.1186/s12967-015-0425-0

**Published:** 2015-02-15

**Authors:** Rui Ma, Yili Feng, Shuang Lin, Jiang Chen, Hui Lin, Xiao Liang, Heming Zheng, Xiujun Cai

**Affiliations:** Department of Surgery, Zhejiang University Hospital, Zhejiang University, Hangzhou, Zhejiang 310027 China; Department of General Surgery, Institute of Minimally Invasive Surgery, Sir Run Run Shaw Hospital, College of Medicine, Zhejiang University, Hangzhou, Zhejiang 310016 China

**Keywords:** Breast cancer, Liver metastasis, Microenvironment

## Abstract

Liver metastasis is a frequent occurrence in patients with breast cancer; however, the available treatments are limited and ineffective. While liver-specific homing of breast cancer cells is an important feature of metastasis, the formation of liver metastases is not random. Indeed, breast cancer cell factors contribute to the liver microenvironment. Major breakthroughs have been achieved recently in understanding breast cancer liver metastasis (BCLM). The process of liver metastasis consists of multiple steps and involves various factors from breast cancer cells and the liver microenvironment. A further understanding of the roles of breast cancer cells and the liver microenvironment is crucial to guide future work in clinical treatments. In this review we discuss the contribution of breast cancer cells and the liver microenvironment to liver metastasis, with the aim to improve therapeutic efficacy for patients with BCLM.

## Introduction

Breast cancer is the leading cause of cancer-related deaths in female patients worldwide [1]. Breast cancer has been divided into at least five subtypes, as follows: luminal A, luminal B, human epithelial growth receptor type 2 (HER-2), basal-like, and claudin-low [2]. It has been reported that the 5-year survival rate for primary breast cancer is 99%. Nevertheless, approximately one-third of breast cancer patients will present with distant non-nodal metastases, and the 5-year survival rate decreases to 23% once distant metastases have developed [3]. Breast cancer mainly metastasizes to the bony skeleton, lungs, liver, and brain via the circulation; the liver is a common metastatic site for solid cancers and represents the third most common site for breast cancer [4]. If breast cancer liver metastasis (BCLM) is left untreated, the survival time is only 4–8 months [5]. Current treatments for BCLM are based on a strategy of systemic chemotherapy, endocrine- or HER2-targeted therapy (depending on estrogen receptor [ER], progesterone receptor [PR], and HER-2 status), and palliative therapy, such as radiation [6,7]. Nevertheless, it is important to note that some patients exhibit *de novo* resistance or eventually become resistant to endocrine therapy [8]. Moreover, a poor response to chemotherapy accounts for much of the high mortality in patients with BCLM [9].

The formation and growth of breast cancer cells in the liver is a complex process. The most widely accepted model for metastasis is the “seed and soil” hypothesis, which was postulated by Stephen Paget in 1889 [10]. Paget speculated that organ metastases form merely when the seed (disseminated tumor cells) and soil (secondary organ) are compatible [10,11]. Semenza separated the process of blood vessel metastasis of breast cancer into the following steps: intravasation, circulation, margination, extravasation, and colonization [12]. The molecular mechanisms underlying breast cancer metastasis have been reported for breast cancer dissemination to the lungs and bone [13-20]; however, the molecular mechanisms for liver metastasis have not been completely described [21]. Moreover, the hepatic microenvironment and liver sinusoidal structure is crucial for the initial arrest of breast cancer and progression within the liver. Thus, exploring the mechanisms underlying liver metastasis in breast cancer patients is essential for developing more effective therapies. A further understanding of the roles of breast cancer cells and the liver microenvironment in early breast cancer metastasis is crucial for the development of effective BCLM therapies [10,22]. Therefore, this review will discuss the molecular mechanisms underlying liver metastasis of breast cancer to guide future work in clinical treatments.

## Factors associated with breast cancer cell metastasis to the liver

### Inflammatory factors

Several studies have shown that the inflammatory response correlates with the liver metastatic potential of some tumors [23,24]. The inflammatory factor, TNF-α, can trigger the expression of E-selectin in endothelial cells, including liver sinusoidal endothelium cells [25,26]. It has been reported that breast cancer cells have the ability to initiate an inflammatory cascade, which increases adhesiveness to liver sinusoidal endothelium cells, similar to that induced by colorectal and lung cancers [27]. Although the process of tumor cell attachment to the endothelium during metastasis is multifactorial, the production of TNF-α-induced endothelial E-selectin in tumor cells appears to be a key step in the BCLM process [28]. Asgeirsson et al. [29] reported that induction of IL-6 decreased cell adhesion in three breast cancer cell lines, and was associated with reduced E-cadherin expression. Moreover, patients with breast cancer liver metastases had significantly higher IL-6 levels. Therefore, it appears that breast cancer cells create a pro-inflammatory microenvironment which triggers adhesion and invasion of tumor cells into the liver by secreting a number of cytokines.

### Chemokines and chemokine receptors

Breast cancer cells express several chemokine receptors that initiate liver metastasis, of which C-X-C Chemokine Receptor type 4 (CXCR4) is the most common. Moreover, the ligand of CXCR4, stromal-derived factor 1-α (SDF-1, CXCL12), is also highly expressed in the liver [30], indicating that the CXCL12/CXCR4 interaction might contribute to BCLM. It has also been reported that CXCR4 plays an important role in modulating breast-to-liver metastasis through integrin–adhesion-receptor signaling [31]. The availability of chemokine receptors on tumor cell surfaces, the presence of specific ligands within the microenvironment of potential target organs, and the suitability of the extracellular matrix (ECM) composition appear to be required for successful extravasation of breast cancer cells in the early metastatic process [32]. Furthermore, expression of CXCR4 increases the risk of metastasis to the liver in patients with axillary node-positive primary breast cancer [33]. In tumor-bearing mice, CC chemokine ligand 2 (CCL2) neutralizing antibodies inhibit the growth and liver metastases of primary breast cancer by reducing cell proliferation, survival, and tumor-associated macrophage (TAM) recruitment. These results have also revealed that CCL2 can enhance primary breast cancer liver metastasis in a TAM-dependent manner [34]. Stormes et al. [35] strongly support the notion that inhibition of tumor-derived CCL5 can inhibit the capability of liver metastasis in breast cancer cells. Similar analyses have shown that the release of CCL5 by cells of the tumor microenvironment promotes the metastatic spread to the liver of breast cancer cells [36]. Moreover, Mi et al. [37] have demonstrated that induced mesenchymal stromal cells (MSCs) produce CCL5, and significantly promote breast cancer cell migration to the liver *in vivo* and *in vitro*. Therefore, some chemokine receptors can enhance breast cancer metastasis to the liver. Porter et al. [38] reported, however, that many chemokines are lost in breast cancer, including CXCL1, CXCL2, CXCL5, CXCL6, CXCL8, CXCL20, CX3CL1, CCL2, and CCL7. Whether or not some of these chemokines are involved in BCLM warrants further study. Taken together, chemokines and their receptors are the regulators involved in the process of BCLM.

### Cell adhesion molecules

#### Cadherins

E-cadherin expression in liver metastatic sites is due to loss of promoter methylation. Breast cancer cells that re-express E-cadherin revert back to an epithelial phenotype [39-42]. In primary cancer, the epithelial-to-mesenchymal transition (EMT) of cancer cells contributes to increased invasion and dissemination [43]. Once cancer cells have seeded the metastatic site, a mesenchymal-to-epithelial transition (MET) occurs, leading to the colonization and growth of metastatic foci [44]. Expression of the cell adhesion molecule, E-cadherin, in breast cancer cells can facilitate breast cancer cell adhesion to hepatocytes for seeding in the liver [9,43]. Moreover, some results have shown that breast cancer cells that express E-cadherin are able to form liver metastases, while E-cadherin-negative cancer cells merely form primary tumors [45,46]. Intriguingly, Chao et al. [9] showed that the liver microenvironment can induce breast cancer cells to re-express E-cadherin and cause MET. This phenotypic change has the potential to alter cell behavior, and thus may be a critical step for cells to survive at metastatic sites within the liver. Take together, these results suggest that re-expression of E-cadherin, accompanied by a partial MET in the liver, increases post-extravasation survival of metastatic cancer cells and may help to elucidate why chemotherapy commonly fails to treat BCLM.

Previous studies have shown that N-cadherin is up-regulated in more invasive and less differentiated breast cancer cell lines that lack E-cadherin expression [47,48]. The study by Hazan et al. [49] showed that breast cancer expressing N-cadherin grew slower, on average, and the two clones with the highest levels of N-cadherin formed liver metastases in almost all mice injected with breast cancer cells. In addition, N-cadherin activates a metastatic signaling pathway coordinated by fibroblast growth factor receptor (FGFR) and matrix metalloproteinase-9 (MMP-9) to overcome the suppressive effects regulated by E-cadherin. Thus, these studies provide a basis for N-cadherin-mediated liver metastasis of breast cancer, whereas E-cadherin suppresses liver metastases, and offer new insights into the diagnostic or therapeutic applications for BCLM.

#### Integrin complexes

Heterodimeric transmembrane receptors of the integrins family are important components of the ECM. Eighteen α-subunits and 8 β-subunits have been characterized and form 24 different integrins [50], including laminin (LN), collagen (Col), fibrinogen, and vitronectin (VN) [51]. Each integrin heterodimer binds to a specific ligand within the ECM. By using bidirectional “outside-in” and “inside-out” signaling, integrin complexes regulate multiple biological processes, such as adhesion, apoptosis, proliferation, differentiation, migration, invasion, and metastasis [52]. It has been reported that use of an antagonist of α2β1 complexes can reduce the extravasation of colorectal and hepatocellular carcinoma cell lines into the liver and micrometastic sites [53]. Moreover, the functional integrin complexes (fibronectin and collagen IV receptors) are recruited, assembled, and thus increased on the cell surface in liver metastatic breast cancer cells. Neutralizing antibody targeting α5β1 or α2β1 complexes can block claudin-2-mediated adhesion to fibronectin and type IV collagen, and reduce the ability of breast cancer cells to metastasize to the liver [54]. Therefore, α2β1 or α5β1 complexes can promote the ability of breast cancer cells to metastasize to the liver, at least partially, via the claudin-2 signaling pathway.

#### Ig-SF

Epithelial cell adhesion molecule (EpCAM/CD326), also a transmembrane protein, plays a variety of roles in cell proliferation, adhesion, migration, and tissue maintenance [55,56]. EpCAM is expressed at low levels in luminal epithelial cells of benign breast tissues. Nevertheless, EpCAM is overexpressed in many carcinomas, including breast cancer; a recent study suggested that p53 dysfunction may serve to explain this phenomenon [57]. In patients with node-positive primary breast cancer, elevated EpCAM expression correlates with diminished overall survival [58,59], suggesting that overexpression of EpCAM promotes cancer progression and metastasis. Litvinov et al. [60,61] also found that EpCAM can act as an antagonist impairing E-cadherin function via disruption of the α-catenin/F-actin link, and loosening tight cell-cell adhesions. In addition, EpCAM is highly expressed in MBCs, including the sites of liver metastases, compared to unmatched, surgically resected primary breast cancers [61]. Therefore, EpCAM might serve as a promising therapeutic target for BCLM.

#### CD44

It has been reported that cancer stem cells (CSCs) within tumors have cancer-initiating potential and metastatic capability [62]. High levels of CD44 expressed by CSCs are believed to be involved in adhesion, invasion, apoptosis resistance, and metastasis [63-65]. Moreover, breast cancer cells expressing high levels of CD44 and low levels of CD24 maintain stemness properties [62,66]. Erin et al. [67] found that CD44 expression was highest in cells that metastasized to the liver, and liver tropism of breast cancer is driven by CSCs. In addition, it has been demonstrated that serum CD44 v5 and v6 are released by breast cancer cells and increased CD44 v6 serum levels are preferentially detected in patients with liver metastases [68,69]. Furthermore, Ouhtit et al. [70] showed that CD44 promotes breast tumor invasion and metastasis to the liver. Together, high levels of CD44 expressed by CSCs and breast cancer cells are significantly correlated with BCLM.

Based on the above results, it appears that cell adhesion molecules play important roles in BCLM (Table 1); however, studies involving cell adhesion molecules that effect BCLM is still limited, and uncovering the mechanisms would provide novel therapeutic approaches in drug design and cancer therapy.

**Table 1:** **Cell adhesion molecules (CAMs) involved in breast cancer liver metastasis**

**CAM family**	**Expression in primary cancer**	**Role**	**Expression in BCLM**	**Role**
**Cadherins**				
E-cadherin	↓Breast cancer cells	Adhesion, invasion, EMT	↑Breast cancer cells [9,43,45,46]	MET
N-cadherin	↑Breast cancer cells,	Angiogenesis, EMT	↑Breast cancer cells [49]	MET
	↑Endothelium cells			
**Integrin complexes**				
α2β1	↓Breast cancer cells,	Adhesion, migration, invasion, angiogenesis	↑Breast cancer cells [54]	liver metastasis, tumor angiogenesis
	↑Endothelium cells			
α5β1	↑Breast cancer cells,	Adhesion, migration, invasion	↑Breast cancer cells [54]	liver metastasis, tumor angiogenesis
	↑Endothelium cells			
**Ig-SF**				
EpCAM	↑Breast cancer cells	Migration, invasion	↑Breast cancer cells [61]	liver metastasis
**CD44**	↓Breast cancer cells (CD44s);	adhesion, invasion	↑Breast cancer cells [67,68]	Liver metastasis
	↑Breast cancer cells (CD44v3, CD44v4, CD44v5, CD44v6)			

Note: ↑, up-regulation; ↓, down-regulation; EMT, epithelial to mesenchymal transition; MET, mesenchymal to epithelial reverting transition.

### Role of claudins

In normal epithelia, claudins are key transmembrane proteins within the tight junction complex that participate in homo- and hetero-typic interactions between adjacent cells [71]. The roles of claudin proteins in breast cancer progression are complicated, and claudin-2 expression is down-regulated compared with normal tissues. Moreover, decreased levels of claudin-2 are observed in high-grade breast cancer, suggesting that claudin-2 plays a suppressive role in breast cancer [72]. Moreover, Kimbung et al. [73] reported that claudin-2 is a prognostic biomarker that not only predicts the likelihood of a breast cancer recurrence, but more interestingly, the metastatic potential of breast cancer to the liver. Furthermore, Tabariès et al. [54,74] demonstrated that elevated levels of claudin-2 expression, or selection for pre-existing claudin-2-positive breast cancer cells within liver metastases may serve to enhance the survival of breast cancer cells by promoting interactions between the tumor cell and resident hepatocytes. Claudin-2 mediates breast cancer metastasis to the liver, at least partially, by enhancing adhesion to ECM proteins, such as fibronectin and type IV collagen, which are abundant in the liver. Immunohistochemical analyses have shown that claudin-2 is detected in all liver metastases and weakly expressed in primary human breast cancers. Take together, these results revealed novel roles of claudin-2 in promoting breast cancer adhesion to the ECM and breast cancer metastasis to the liver.

The up-regulation of claudin-3 and −4 is correlated with poor prognosis and the breast cancer basal-like subtype [75-77]. A recent study demonstrated that the loss of claudin-4 and −7 promoted liver metastasis of breast cancer cells in Balb-c mice [78]. We speculate that the discrepant roles of claudin-4 with poor prognosis in different studies might be due to different subtypes or species. Intriguingly, a ‘claudin-low’ subtype of breast cancer has recently been identified in primary human breast cancers. This subtype possesses features similar to, but distinct from the breast cancer basal subtype defined previously [79]; the breast cancer claudin-low and basal subtypes are functionally coupled by EMT characteristics, enhanced cancer stem cell–like features, and resistance to chemotherapy [80-84]. Nevertheless, the patterns of expression of claudins in the claudin-low subtype of breast cancer have not been described.

In summary, the above findings imply that claudin-2, −4, and −7 are all necessary and sufficient for the ability of breast cancer cells to colonize and grow in the liver. It is important to delineate the mechanism underlying preferential metastasis of breast cancer to the liver.

### Breast cancer subtypes

It has been reported that five major subtypes of breast cancer have different abilities to metastasize to distant organs, and share pathways with the preferred metastatic sites. Patients with bone relapses have the luminal subtypes of breast cancer most frequently. The HER-2 subtype may metastasize to bone via processes that differ from the luminal subtypes. Moreover, the basal subtype often metastasizes to the brain and lungs. However, it fails to reach statistical significance in patients with liver relapse [85]. Rodriguez-Pinilla et al. [86] reported that the basal-like subtype metastasizes more frequently to the lungs and other visceral organs, such as the brain and liver, but no bone metastases were detected. Moreover, recent studies [87-89] in patients from eastern Europe, Asia, and the US have suggested a high incidence of brain metastases arising from basal-like tumors (ER-/PR-/HER2-, and usually identified as “triple-negative” breast cancer [TNBC]) [90], but it has been reported that p53-negative TNBC has an increased tendency to develop lung metastases [91]. Furthermore, Duan et al. [92] showed that the breast cancer subtype is an independent prognostic predictor for patients with breast cancer metastases to the liver. Survival after liver metastases arising from TNBC is 21 months compared to 30, 32, and 41 months for patients with the HER-2, luminal B, and luminal A subtypes; liver metastases from TNBC has the worst prognosis. Therefore, novel agents controlling liver metastases in patients with TNBC are needed. Taken together, different subtypes of breast cancer have preferred metastatic organs; however, no conclusive evidence exists to mechanistically link any specific subtype to BCLM development.

## Factors associated with the liver microenvironment

### Hypoxia-inducible factor-regulated genes

Hypoxia-inducible factors (HIFs) activate the transcription of target genes that are involved in many aspects of breast cancer progression, such as angiogenesis, metabolic reprogramming, local tissue invasion, and metastasis [12,93]. It has been reported that HIFs not only activate lysyl oxidase (LOX) directly to inhibit liver metastases, but osteopontin (OPN), vascular endothelial growth factor (VEGF), and TWIST promote BCLM (Table 2). It has been suggested that some of the hypoxia-inducible factor-regulated genes contribute to BCLM [37,69,94,95].

**Table 2:** **Hypoxia-inducible factor (HIF)-regulated genes in breast cancer liver metastasis**

**Gene**	**HIF regulation**	**Role in BCLM**
LOX [94]	HIF-1 and HIF-2	Inhibition
OPN [37,69]	HIF-1 (HIF-2 not tested)	Promotion
Twist [95,116]	HIF-1 (HIF-2 not tested)	Promotion
VEGF [95]	HIF-1 and HIF-2	Promotion

#### LOX

LOX is an amine oxidase that contributes to the formation of the ECM. LOX is secreted by fibrinogenic cells and residues in collagen and elastin to maintain the structural stabilization of the ECM [96]. Of note, LOX expression has paradoxical roles in tumor suppression and tumor progression, depending on cellular location, type, and transformation status [96-101]. Erler et al. [94] proposed that hypoxia-induced LOX has a key function in the metastasis of breast cancer cells. Although no marked inhibition effects of LOX on primary breast cancer growth was observed, Erler et al. [94] found significant effects on growth in metastatic sites within the liver. These data indicate that the effects of LOX on cell adhesion, migration, invasion, and three-dimensional growth are more crucial for liver metastatic growth than primary breast cancers. This study may provide mechanistic evidence for hypoxia-driven liver metastases and support the therapeutic target of LOX in the prevention and treatment of BCLM.

#### OPN

HIF-induced OPN is a secreted phosphoprotein functioning as a cell attachment protein by binding two cell adhesion molecules (αvβ3 integrin and CD44) [102-104]. It has been reported that OPN is overexpressed in tumors and elevated serum OPN levels are associated with advanced metastatic cancer [105-108]. Gain- and loss-of-function assays have demonstrated the critical role for OPN in tumor metastases of colon, liver, and breast cancers [69]. Mi et al. [37] revealed that MSCs extracted from metastatic sites exhibit significantly increased expression of cancer-associated fibroblast (CAF) markers, such as α-smooth muscle actin (α-SMA), tenascin-c, CXCL12, and fibroblast-specific protein (FSP)-1. OPN expression promotes tumor growth and metastasis by activating the expression of CCL5, MMPs, and CAF in MSCs. Thus, the transformation of MSCs to CAF can be mediated by OPN in the tumor microenvironment. In addition, the current study showed that the expression of CAF markers was also significantly increased in the liver metastases sites. These findings suggest a novel mechanism by which OPN affects BCLM by transforming MSCs into a CAF.

#### VEGF and TWIST

Cancer cells express a number of angiogenic factors. VEGFs are the key mediator of neovascularization in tumors [109]. Inhibition of VEGF-A/VEGFR-2 and VEGF-C/VEGFR-3 signals has been shown to suppress breast cancer progression and lung metastases [110]. Chien et al. [111] found that inhibition of VEGFR/FGFR kinases drastically reduce the formation of liver metastases and decreased primary breast cancer growth. TWIST is a basic helix-loop-helix transcription factor. TWIST mainly regulates gastrulation and mesoderm specification [112,113]. Recently, TWIST has been shown to play an important role in mediating cancer metastasis [114,115]. TWIST is a downstream target of HIF-1 and has an important role in metastatic phenotypes induced by hypoxia or overexpression of HIF-1α in breast cancer cell lines (MCF-7). HIF-1α promotes hypoxia-induced breast cancer progression and metastasis through the direct activation of TWIST expression [116].

In a previous report, inhibition of HIF-1α by 2-benzoyl-3-phenyl-6, 7-dichloroquinoxaline 1, 4-dioxide (DCQ) was shown to block VEGF secretion and invasion in MCF-7 and led to the inhibition of TWIST expression in MDA-MB-231. DCQ exhibits robust anti-tumor activity in MDA-MB-231 breast cancer mouse xenografts. Also, DCQ reduces metastatic dissemination to the liver, leading to prolonged animal survival [95]. Therefore, HIF-1α can promote BCLM through VEGF and TWIST signaling pathways.

### Role of the vasculature might be essential for liver metastasis

Vermeulen et al. [117] reported that colorectal cancer metastases to the liver grow according to three different histologic patterns, termed ‘pushing’, ‘replacement’, and ‘desmoplastic’ growth patterns. The results of subsequent studies [118,119] involving liver metastases of colorectal and breast cancers showed that the different grow patterns have different angiogenic properties. The replacement pattern grows by co-opting the stroma, without induction of hypoxia or angiogenesis and thus minimal perturbation of the liver architecture; however, the pushing and desmoplastic patterns grow in an angiogenic fashion, which is at least in part hypoxia-driven. Furthermore, the pattern of replacement growth in a non-angiogenic process is even more prevalent in breast cancer than colorectal cancer, and the process induces neither hypoxia nor vascular leakage.

Martin et al. [120] has shown that the majority of early metastatic foci in the liver contain few cells, even 12 days after breast cancer cell injection. Only a few foci were able to develop into micrometastatic lesions with a patent vasculature, thus suggesting that lesions that utilize an existent patent blood supply can thrive in the liver microenvironment, while the remaining foci without a vascular supply remain dormant in the liver. Naumov et al. [121] suggested that tumors are dependent on angiogenesis for progressive growth and remain harmless to the organism at the non-angiogenic dormant stage. Furthermore, the expansion of tumor mass is associated with recruiting endothelial cells after the cancer tissues undergo a switch from a non-angiogenic dormant phenotype to the angiogenic phenotype [122].

Therefore, we speculate that breast cancer metastases to the liver grow mainly by the replacement pattern with a non-angiogenic process in the initial stage, and the vasculature is crucial for thriving in the sites of liver metastases in the late stage.

### Role of sinusoidal capillaries

Previous studies have reported that the initial arrest of cancer cells in the sinusoids of the liver is restricted by the sizes of cancer cells [123,124]. Haier et al. [125] has determined that tumor cells adhere to sinusoidal capillaries, the internal diameter of which is larger than the tumor cells. Unique structural features of liver, including the existence of a fenestrated endothelium (sinusoidal endothelium) and lack of an organized sub-endothelial basement membrane, have a great impact on the interactions between breast cancer cells and the liver microenvironment. Of great interest, the fenestrated endothelium controls liver-specific microvascular exchange and impacts the ability of cells to transmigrate through the vessels into the liver [126]. Moreover, previous studies have revealed that breast cancer cells extend cellular projections through the fenestrated endothelium into the space of Disse on seeding the liver, which makes direct contact with hepatocytes [127]. In addition, Martin et al. [120] has found that breast cancer cells are bound to vessels with clear vascular labeling in the sites of liver metastases. Thus, sinusoidal capillaries play a significant role in the initial arrest of breast cancer.

### Changes in hormonal receptor status and HER-2

ER, PR, and HER-2 status is essential in determining the use and evaluating the effect of adjuvant hormone therapy, molecular targeted therapy, and even chemotherapy. Koo et al. [128] conducted a study to assess the status of ER, PR, and HER-2 in primary and metastatic breast cancers and determined the relationship between ER, PR, and HER-2 and organ-specific metastases of breast cancer. The data showed that ER+ or PR+/HER-2- (luminal A) subtypes were predominant in the sites of liver metastases (75.0%). Increased phosphorylation of HER-2 appears to be extremely important for the establishment of breast cancer liver metastases [129]. Moreover, a high serum HER2 level or lack of ERs independently doubles the relative risk of progression and mortality [130].

Nevertheless, the ER, PR, and HER-2 status between primary breast cancers and liver metastatic foci can be changed after treatment, but are stable in most cases during the liver metastatic process [131]. Botteri et al. [132] conducted a retrospective study of patients with BCLM and found a positive relationship between liver biopsy findings and survival in patients with early metastases. Moreover, another study showed that biopsies of metastases are useful for the reassessment of the metastatic sites to define a more effective treatment strategy for patients with BCLM [133]. Thus, the ER, PR, and HER-2 status needs to be reassessed by biopsy when liver metastases occur.

## A model for breast cancer liver metastasis

**Figure 1:**
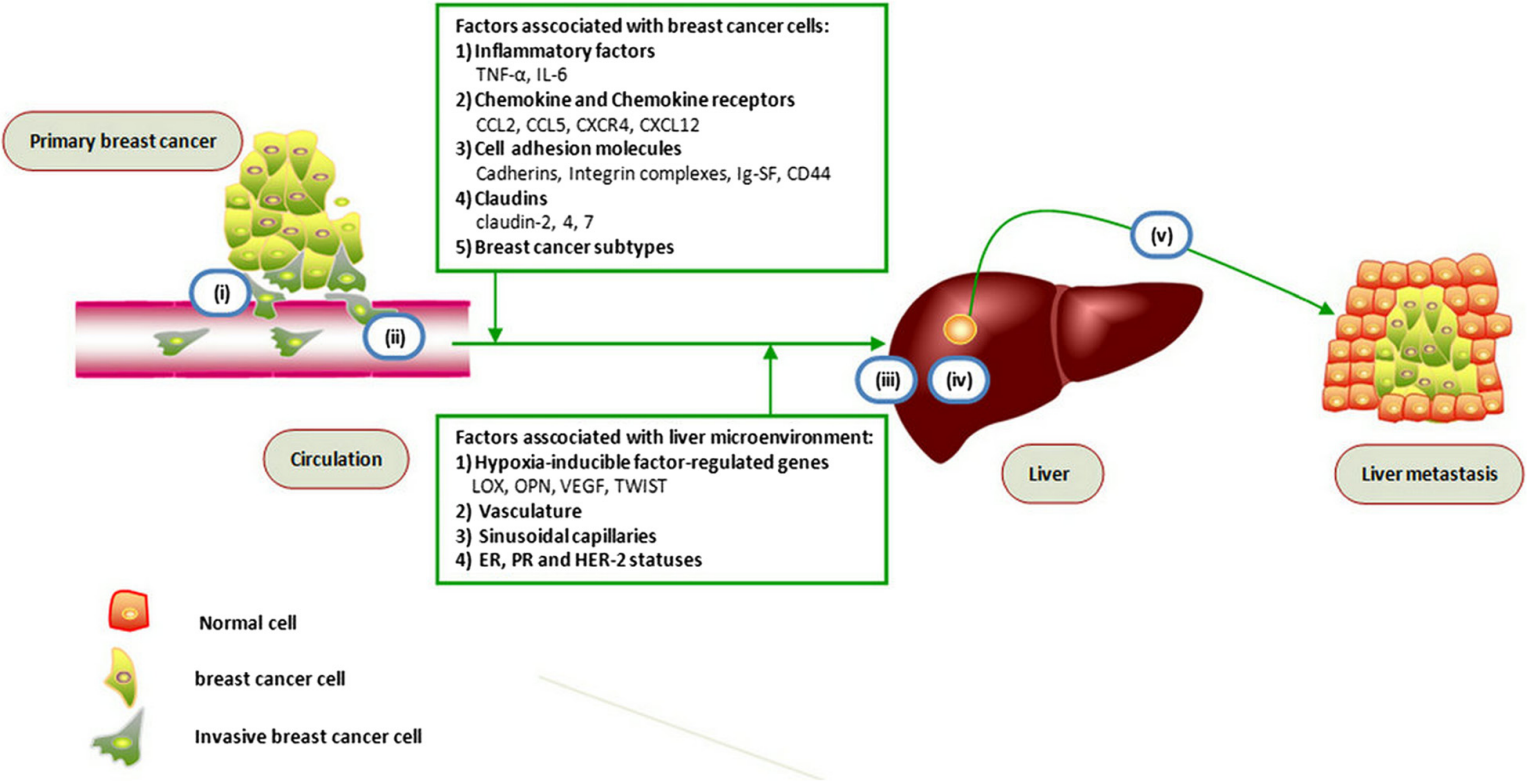
**A model for breast cancer liver metastasis.** (i) intravasation: invasive breast cancer cell invades through the endothelium of a tumor blood vessel into circulation; (ii) circulation: breast cancer cell survives in the blood vessels without any attachment; (iii) margination: circulating breast cancer cell arrests at the site of liver by adhering to the sinusoidal endothelial cell via specific sets of adhesion molecules; (iv) extravasation: the migrated breast cancer cell invades through the endothelial wall of sinusoidal endothelial cell, migrates and finally proliferates in the liver; and (v) colonization: breast cancer cells survive and form a life-threatening focus in liver.

An increasingly sophisticated understanding of breast cancer liver metastasis is emerging. It appears that BCLM is mainly associated with specific subtypes in patients with breast cancer [85,86,92]; however, no direct correlation between subtypes and BCLM has been found. By combining the knowledge from the extant research, we propose a model for breast cancer liver metastasis (Figure 1), as follows: (i) intravasation: invasive breast cancer cells invade via the endothelium of a tumor blood vessel into the circulation; (ii) circulation: breast cancer cells survive in the blood vessels and lack of cell-cell or cell-matrix attachments; (iii) margination: CTCs arrest at the liver site by adhering to the sinusoidal endothelial cell via specific sets of adhesion molecules, such as cadherins, integrins, Ig-SF, and CD44; (iv) extravasation: the migrated breast cancer cells invade through the endothelial wall of sinusoidal endothelial cells, migrates, and finally proliferates in the liver (in this process, the diameter of the sinusoidal endothelium and the lack of an organized sub-endothelial basement membrane have a great impact on breast cancer cell migration); and (v) colonization: breast cancer cells survive and form a life-threatening macrometastatic focus in the liver microenvironment by mediating hypoxia-inducible factor-regulated genes (LOX, OPN, VEGF, and TWIST), the status of ER, PR, and HER-2 expression, and angiogenesis for breast cancer cells.

## Conclusions

The process of BCLM includes several steps and is influenced by various factors. Although major breakthroughs have been achieved recently in understanding of BCLM, no effective therapies have been developed. Further understanding of the roles of breast cancer cells and the liver microenvironment will open a new window to guide future work in clinical treatments.

## Abbreviations

BCLM: Breast cancer liver metastasis
CAF: cancer-associated fibroblast
CCL2: CC chemokine ligand 2
Col: Collagen
CSCs: Cancer stem cells
CXCR4: C-X-C Chemokine Receptor type 4
DCQ: 2-benzoyl-3-phenyl-6, 7-dichloroquinoxaline 1, 4-dioxide
ECM: Extracellular matrix
EMT: Epithelial to mesenchymal transition
EpCAM: Epithelial cell adhesion molecule
ER: Estrogen receptor
FGFR: Fibroblast growth factor receptor
FSP: Fibroblast-specific protein
HER2: Human epidermal growth factor receptor-2
HIF: Hypoxia-inducible factor
LN: Laminin
LOX: Lysyl oxidase
MET: Mesenchymal to epithelial transition
MMP-9: Matrix metalloproteinases 9
MSC: Mesenchymal stromal cell
OPN: Osteopontin
PR: Progesterone receptor
TAM: Tumor-associated macrophage
TNBC: “triple-negative” breast cancer
VEGF: Vascular endothelial growth factor
VN: Vitronectin
α-SMA: α-smooth muscle actin

## References

[1] Jemal A, Center MM, DeSantis C, Ward EM (2010). Global patterns of cancer incidence and mortality rates and trends. Cancer Epidemiol Biomarkers Prev.

[2] Perou CM, Sorlie T, Eisen MB, van de Rijn M, Jeffrey SS, Rees CA (2000). Molecular portraits of human breast tumours. Nature.

[3] Siegel R, Naishadham D, Jemal A (2012). Cancer statistics, 2012. CA Cancer J Clin.

[4] Hess KR, Varadhachary GR, Taylor SH, Wei W, Raber MN, Lenzi R (2006). Metastatic patterns in adenocarcinoma. Cancer.

[5] Adam R, Aloia T, Krissat J, Bralet MP, Paule B, Giacchetti S (2006). Is liver resection justified for patients with hepatic metastases from breast cancer?. Ann Surg.

[6] Diamond JR, Finlayson CA, Borges VF (2009). Hepatic complications of breast cancer. Lancet Oncol.

[7] Hortobagyi GN (2005). Trastuzumab in the treatment of breast cancer. N Engl J Med.

[8] Miller WR (2010). Aromatase inhibitors: prediction of response and nature of resistance. Expert Opin Pharmacother.

[9] Chao Y, Wu Q, Shepard C, Wells A (2012). Hepatocyte induced re-expression of E-cadherin in breast and prostate cancer cells increases chemoresistance. Clin Exp Metastasis.

[10] Paget S (1989). The distribution of secondary growths in cancer of the breast. 1889. Cancer Metastasis Rev.

[11] Fidler IJ (2003). The pathogenesis of cancer metastasis: the ‘seed and soil’ hypothesis revisited. Nat Rev Cancer.

[12] Semenza GL (2013). Cancer-stromal cell interactions mediated by hypoxia-inducible factors promote angiogenesis, lymphangiogenesis, and metastasis. Oncogene.

[13] Chiu HW, Yeh YL, Wang YC, Huang WJ, Chen YA, Chiou YS, et al. Suberoylanilide hydroxamic acid, an inhibitor of histone deacetylase, enhances radiosensitivity and suppresses lung metastasis in breast cancer in vitro and in vivo. PLoS One. 2013;8:e76340. 10.1371/journal.pone.0076340 PMC3794942 24130769

[14] Gao H, Chakraborty G, Lee-Lim AP, Mo Q, Decker M, Vonica A (2012). The BMP inhibitor Coco reactivates breast cancer cells at lung metastatic sites. Cell.

[15] Jiang J, Thyagarajan-Sahu A, Loganathan J, Eliaz I, Terry C, Sandusky GE (2012). BreastDefend prevents breast-to-lung cancer metastases in an orthotopic animal model of triple-negative human breast cancer. Oncol Rep.

[16] Loganathan J, Jiang J, Smith A, Jedinak A, Thyagarajan-Sahu A, Sandusky GE (2014). The mushroom Ganoderma lucidum suppresses breast-to-lung cancer metastasis through the inhibition of pro-invasive genes. Int J Oncol.

[17] Minn AJ, Gupta GP, Siegel PM, Bos PD, Shu W, Giri DD (2005). Genes that mediate breast cancer metastasis to lung. Nature.

[18] Akhtari M, Mansuri J, Newman KA, Guise TM, Seth P (2008). Biology of breast cancer bone metastasis. Cancer Biol Ther.

[19] Suva LJ, Griffin RJ, Makhoul I (2009). Mechanisms of bone metastases of breast cancer. Endocr Relat Cancer.

[20] Zhang Y, Ma B, Fan Q (2010). Mechanisms of breast cancer bone metastasis. Cancer Lett.

[21] Lu X, Kang Y (2007). Organotropism of breast cancer metastasis. J Mammary Gland Biol Neoplasia.

[22] Price JE, Naito S, Fidler IJ (1988). Growth in an organ microenvironment as a selective process in metastasis. Clin Exp Metastasis.

[23] St Hill CA (2011). Interactions between endothelial selectins and cancer cells regulate metastasis. Front Biosci.

[24] Khatib AM, Fallavollita L, Wancewicz EV, Monia BP, Brodt P (2002). Inhibition of hepatic endothelial E-selectin expression by C-raf antisense oligonucleotides blocks colorectal carcinoma liver metastasis. Cancer Res.

[25] Auguste P, Fallavollita L, Wang N, Burnier J, Bikfalvi A, Brodt P (2007). The host inflammatory response promotes liver metastasis by increasing tumor cell arrest and extravasation. Am J Pathol.

[26] Brodt P, Fallavollita L, Bresalier RS, Meterissian S, Norton CR, Wolitzky BA (1997). Liver endothelial E-selectin mediates carcinoma cell adhesion and promotes liver metastasis. Int J Cancer.

[27] Khatib AM, Auguste P, Fallavollita L, Wang N, Samani A, Kontogiannea M (2005). Characterization of the host proinflammatory response to tumor cells during the initial stages of liver metastasis. Am J Pathol.

[28] Eichbaum C, Meyer AS, Wang N, Bischofs E, Steinborn A, Bruckner T (2011). Breast cancer cell-derived cytokines, macrophages and cell adhesion: implications for metastasis. Anticancer Res.

[29] Asgeirsson KS, Olafsdottir K, Jonasson JG, Ogmundsdottir HM (1998). The effects of IL-6 on cell adhesion and e-cadherin expression in breast cancer. Cytokine+.

[30] Muller A, Homey B, Soto H, Ge N, Catron D, Buchanan ME (2001). Involvement of chemokine receptors in breast cancer metastasis. Nature.

[31] Furusato B, Mohamed A, Uhlen M, Rhim JS (2010). CXCR4 and cancer. Pathol Int.

[32] Wendel C, Hemping-Bovenkerk A, Krasnyanska J, Mees ST, Kochetkova M, Stoeppeler S (2012). CXCR4/CXCL12 participate in extravasation of metastasizing breast cancer cells within the liver in a rat model. PLoS One.

[33] Andre F, Cabioglu N, Assi H, Sabourin JC, Delaloge S, Sahin A (2006). Expression of chemokine receptors predicts the site of metastatic relapse in patients with axillary node positive primary breast cancer. Ann Oncol.

[34] Hembruff SL, Jokar I, Yang L, Cheng N (2010). Loss of transforming growth factor-beta signaling in mammary fibroblasts enhances CCL2 secretion to promote mammary tumor progression through macrophage-dependent and -independent mechanisms. Neoplasia.

[35] Stormes KA, Lemken CA, Lepre JV, Marinucci MN, Kurt RA (2005). Inhibition of metastasis by inhibition of tumor-derived CCL5. Breast Cancer Res Treat.

[36] Soria G, Ben-Baruch A (2008). The inflammatory chemokines CCL2 and CCL5 in breast cancer. Cancer Lett.

[37] Mi Z, Bhattacharya SD, Kim VM, Guo H, Talbot LJ, Kuo PC (2011). Osteopontin promotes CCL5-mesenchymal stromal cell-mediated breast cancer metastasis. Carcinogenesis.

[38] Porter DA, Krop IE, Nasser S, Sgroi D, Kaelin CM, Marks JR (2001). A SAGE (serial analysis of gene expression) view of breast tumor progression. Cancer Res.

[39] Hugo H, Ackland ML, Blick T, Lawrence MG, Clements JA, Williams ED (2007). Epithelial–mesenchymal and mesenchymal–epithelial transitions in carcinoma progression. J Cell Physiol.

[40] Brabletz T, Jung A, Reu S, Porzner M, Hlubek F, Kunz-Schughart LA (2001). Variable beta-catenin expression in colorectal cancers indicates tumor progression driven by the tumor environment. Proc Natl Acad Sci U S A.

[41] Chaffer CL, Brennan JP, Slavin JL, Blick T, Thompson EW, Williams ED (2006). Mesenchymal-to-epithelial transition facilitates bladder cancer metastasis: role of fibroblast growth factor receptor-2. Cancer Res.

[42] Hudson LG, Zeineldin R, Stack MS (2008). Phenotypic plasticity of neoplastic ovarian epithelium: unique cadherin profiles in tumor progression. Clin Exp Metastasis.

[43] Thiery JP, Acloque H, Huang RY, Nieto MA (2009). Epithelial-mesenchymal transitions in development and disease. Cell.

[44] Polyak K, Weinberg RA (2009). Transitions between epithelial and mesenchymal states: acquisition of malignant and stem cell traits. Nat Rev Cancer.

[45] Lou Y, Preobrazhenska O, auf dem Keller U, Sutcliffe M, Barclay L, McDonald PC (2008). Epithelial-mesenchymal transition (EMT) is not sufficient for spontaneous murine breast cancer metastasis. Dev Dyn.

[46] Wang HH, McIntosh AR, Hasinoff BB, Rector ES, Ahmed N, Nance DM (2000). B16 melanoma cell arrest in the mouse liver induces nitric oxide release and sinusoidal cytotoxicity: a natural hepatic defense against metastasis. Cancer Res.

[47] Hazan RB, Kang L, Whooley BP, Borgen PI (1997). N-cadherin promotes adhesion between invasive breast cancer cells and the stroma. Cell Adhes Commun.

[48] Kern FG, McLeskey SW, Zhang L, Kurebayashi J, Liu Y, Ding IY (1994). Transfected MCF-7 cells as a model for breast-cancer progression. Breast Cancer Res Treat.

[49] Hazan RB, Phillips GR, Qiao RF, Norton L, Aaronson SA (2000). Exogenous expression of N-cadherin in breast cancer cells induces cell migration, invasion, and metastasis. J Cell Biol.

[50] van der Flier A, Sonnenberg A (2001). Function and interactions of integrins. Cell Tissue Res.

[51] Rathinam R, Alahari SK (2010). Important role of integrins in the cancer biology. Cancer Metastasis Rev.

[52] Harburger DS, Calderwood DA (2009). Integrin signalling at a glance. J Cell Sci.

[53] Rosenow F, Ossig R, Thormeyer D, Gasmann P, Schluter K, Brunner G (2008). Integrins as antimetastatic targets of RGD-independent snake venom components in liver metastasis [corrected]. Neoplasia.

[54] Tabaries S, Dong Z, Annis MG, Omeroglu A, Pepin F, Ouellet V (2011). Claudin-2 is selectively enriched in and promotes the formation of breast cancer liver metastases through engagement of integrin complexes. Oncogene.

[55] Trzpis M, McLaughlin PM, de Leij LM, Harmsen MC (2007). Epithelial cell adhesion molecule: more than a carcinoma marker and adhesion molecule. Am J Pathol.

[56] Baeuerle PA, Gires O (2007). EpCAM (CD326) finding its role in cancer. Br J Cancer.

[57] Sankpal NV, Willman MW, Fleming TP, Mayfield JD, Gillanders WE (2009). Transcriptional repression of epithelial cell adhesion molecule contributes to p53 control of breast cancer invasion. Cancer Res.

[58] Gastl G, Spizzo G, Obrist P, Dunser M, Mikuz G (2000). Ep-CAM overexpression in breast cancer as a predictor of survival. Lancet.

[59] Spizzo G, Went P, Dirnhofer S, Obrist P, Simon R, Spichtin H (2004). High Ep-CAM expression is associated with poor prognosis in node-positive breast cancer. Breast Cancer Res Treat.

[60] Litvinov SV, Balzar M, Winter MJ, Bakker HA, Briaire-de Bruijn IH, Prins F (1997). Epithelial cell adhesion molecule (Ep-CAM) modulates cell-cell interactions mediated by classic cadherins. J Cell Biol.

[61] Cimino A, Halushka M, Illei P, Wu X, Sukumar S, Argani P (2010). Epithelial cell adhesion molecule (EpCAM) is overexpressed in breast cancer metastases. Breast Cancer Res Treat.

[62] Al-Hajj M, Wicha MS, Benito-Hernandez A, Morrison SJ, Clarke MF (2003). Prospective identification of tumorigenic breast cancer cells. Proc Natl Acad Sci U S A.

[63] Brown LF, Berse B, Van de Water L, Papadopoulos-Sergiou A, Perruzzi CA, Manseau EJ (1992). Expression and distribution of osteopontin in human tissues: widespread association with luminal epithelial surfaces. Mol Biol Cell.

[64] Zoller M (2011). CD44: can a cancer-initiating cell profit from an abundantly expressed molecule?. Nat Rev Cancer.

[65] Sun H, Jia J, Wang X, Ma B, Di L, Song G (2013). CD44+/CD24- breast cancer cells isolated from MCF-7 cultures exhibit enhanced angiogenic properties. Clin Transl Oncol.

[66] Mani SA, Guo W, Liao MJ, Eaton EN, Ayyanan A, Zhou AY (2008). The epithelial-mesenchymal transition generates cells with properties of stem cells. Cell.

[67] Erin N, Kale S, Tanriover G, Koksoy S, Duymus O, Korcum AF (2013). Differential characteristics of heart, liver, and brain metastatic subsets of murine breast carcinoma. Breast Cancer Res Treat.

[68] Lackner C, Moser R, Bauernhofer T, Wilders-Truschnig M, Samonigg H, Berghold A (1998). Soluble CD44 v5 and v6 in serum of patients with breast cancer. Correlation with expression of CD44 v5 and v6 variants in primary tumors and location of distant metastasis. Breast Cancer Res Treat.

[69] Wai PY, Kuo PC (2004). The role of Osteopontin in tumor metastasis. J Surg Res.

[70] Ouhtit A, Abd Elmageed ZY, Abdraboh ME, Lioe TF, Raj MH (2007). In vivo evidence for the role of CD44s in promoting breast cancer metastasis to the liver. Am J Pathol.

[71] Turksen K, Troy TC (2004). Barriers built on claudins. J Cell Sci.

[72] Kim TH, Huh JH, Lee S, Kang H, Kim GI, An HJ (2008). Down-regulation of claudin-2 in breast carcinomas is associated with advanced disease. Histopathology.

[73] Kimbung S, Kovacs A, Bendahl PO, Malmstrom P, Ferno M, Hatschek T (2014). Claudin-2 is an independent negative prognostic factor in breast cancer and specifically predicts early liver recurrences. Mol Oncol.

[74] Tabaries S, Dupuy F, Dong Z, Monast A, Annis MG, Spicer J (2012). Claudin-2 promotes breast cancer liver metastasis by facilitating tumor cell interactions with hepatocytes. Mol Cell Biol.

[75] Blanchard AA, Skliris GP, Watson PH, Murphy LC, Penner C, Tomes L (2009). Claudins 1, 3, and 4 protein expression in ER negative breast cancer correlates with markers of the basal phenotype. Virchows Arch.

[76] Kulka J, Szasz AM, Nemeth Z, Madaras L, Schaff Z, Molnar IA (2009). Expression of tight junction protein claudin-4 in basal-like breast carcinomas. Pathol Oncol Res.

[77] Lanigan F, McKiernan E, Brennan DJ, Hegarty S, Millikan RC, McBryan J (2009). Increased claudin-4 expression is associated with poor prognosis and high tumour grade in breast cancer. Int J Cancer.

[78] Erin N, Wang N, Xin P, Bui V, Weisz J, Barkan GA (2009). Altered gene expression in breast cancer liver metastases. Int J Cancer.

[79] Herschkowitz JI, Simin K, Weigman VJ, Mikaelian I, Usary J, Hu Z, et al. Identification of conserved gene expression features between murine mammary carcinoma models and human breast tumors. Genome Biol. 2007;8:R76. 10.1186/gb-2007-8-5-r76 PMC1929138 17493263

[80] Creighton CJ, Li X, Landis M, Dixon JM, Neumeister VM, Sjolund A (2009). Residual breast cancers after conventional therapy display mesenchymal as well as tumor-initiating features. Proc Natl Acad Sci U S A.

[81] Creighton CJ, Chang JC, Rosen JM (2010). Epithelial-mesenchymal transition (EMT) in tumor-initiating cells and its clinical implications in breast cancer. J Mammary Gland Biol Neoplasia.

[82] Hennessy BT, Gonzalez-Angulo AM, Stemke-Hale K, Gilcrease MZ, Krishnamurthy S, Lee JS (2009). Characterization of a naturally occurring breast cancer subset enriched in epithelial-to-mesenchymal transition and stem cell characteristics. Cancer Res.

[83] Prat A, Parker JS, Karginova O, Fan C, Livasy C, Herschkowitz JI (2010). Phenotypic and molecular characterization of the claudin-low intrinsic subtype of breast cancer. Breast Cancer Res.

[84] Taube JH, Herschkowitz JI, Komurov K, Zhou AY, Gupta S, Yang J (2010). Core epithelial-to-mesenchymal transition interactome gene-expression signature is associated with claudin-low and metaplastic breast cancer subtypes. Proc Natl Acad Sci U S A.

[85] Smid M, Wang Y, Zhang Y, Sieuwerts AM, Yu J, Klijn JG (2008). Subtypes of breast cancer show preferential site of relapse. Cancer Res.

[86] Rodriguez-Pinilla SM, Sarrio D, Honrado E, Hardisson D, Calero F, Benitez J (2006). Prognostic significance of basal-like phenotype and fascin expression in node-negative invasive breast carcinomas. Clin Cancer Res.

[87] Nam BH, Kim SY, Han HS, Kwon Y, Lee KS, Kim TH (2008). Breast cancer subtypes and survival in patients with brain metastases. Breast Cancer Res.

[88] Heitz F, Harter P, Lueck HJ, Fissler-Eckhoff A, Lorenz-Salehi F, Scheil-Bertram S (2009). Triple-negative and HER2-overexpressing breast cancers exhibit an elevated risk and an earlier occurrence of cerebral metastases. Eur J Cancer.

[89] Niwinska A, Murawska M, Pogoda K (2010). Breast cancer brain metastases: differences in survival depending on biological subtype, RPA RTOG prognostic class and systemic treatment after whole-brain radiotherapy (WBRT). Ann Oncol.

[90] Lin NU, Claus E, Sohl J, Razzak AR, Arnaout A, Winer EP (2008). Sites of distant recurrence and clinical outcomes in patients with metastatic triple-negative breast cancer: high incidence of central nervous system metastases. Cancer.

[91] Gao D, Du J, Cong L, Liu Q (2009). Risk factors for initial lung metastasis from breast invasive ductal carcinoma in stages I-III of operable patients. Jpn J Clin Oncol.

[92] Duan XF, Dong NN, Zhang T, Li Q (2013). The prognostic analysis of clinical breast cancer subtypes among patients with liver metastases from breast cancer. Int J Clin Oncol.

[93] Semenza GL (2013). HIF-1 mediates metabolic responses to intratumoral hypoxia and oncogenic mutations. J Clin Invest.

[94] Erler JT, Bennewith KL, Nicolau M, Dornhofer N, Kong C, Le QT (2006). Lysyl oxidase is essential for hypoxia-induced metastasis. Nature.

[95] Ghattass K, El-Sitt S, Zibara K, Rayes S, Haddadin MJ, El-Sabban M (2014). The quinoxaline di-N-oxide DCQ blocks breast cancer metastasis in vitro and in vivo by targeting the hypoxia inducible factor-1 pathway. Mol Cancer.

[96] Kagan HM, Li W (2003). Lysyl oxidase: properties, specificity, and biological roles inside and outside of the cell. J Cell Biochem.

[97] Csiszar K, Fong SF, Ujfalusi A, Krawetz SA, Salvati EP, Mackenzie JW (2002). Somatic mutations of the lysyl oxidase gene on chromosome 5q23.1 in colorectal tumors. Int J Cancer.

[98] Kirschmann DA, Seftor EA, Fong SF, Nieva DR, Sullivan CM, Edwards EM (2002). A molecular role for lysyl oxidase in breast cancer invasion. Cancer Res.

[99] Kaneda A, Wakazono K, Tsukamoto T, Watanabe N, Yagi Y, Tatematsu M (2004). Lysyl oxidase is a tumor suppressor gene inactivated by methylation and loss of heterozygosity in human gastric cancers. Cancer Res.

[100] Palamakumbura AH, Jeay S, Guo Y, Pischon N, Sommer P, Sonenshein GE (2004). The propeptide domain of lysyl oxidase induces phenotypic reversion of ras-transformed cells. J Biol Chem.

[101] Payne SL, Fogelgren B, Hess AR, Seftor EA, Wiley EL, Fong SF (2005). Lysyl oxidase regulates breast cancer cell migration and adhesion through a hydrogen peroxide-mediated mechanism. Cancer Res.

[102] Denhardt DT, Lopez CA, Rollo EE, Hwang SM, An XR, Walther SE (1995). Osteopontin-induced modifications of cellular functions. Ann N Y Acad Sci.

[103] Denhardt DT, Giachelli CM, Rittling SR (2001). Role of osteopontin in cellular signaling and toxicant injury. Annu Rev Pharmacol Toxicol.

[104] Weber GF, Ashkar S, Cantor H (1997). Interaction between CD44 and osteopontin as a potential basis for metastasis formation. Proc Assoc Am Physicians.

[105] Das R, Mahabeleshwar GH, Kundu GC (2003). Osteopontin stimulates cell motility and nuclear factor kappaB-mediated secretion of urokinase type plasminogen activator through phosphatidylinositol 3-kinase/Akt signaling pathways in breast cancer cells. J Biol Chem.

[106] Fedarko NS, Jain A, Karadag A, Van Eman MR, Fisher LW (2001). Elevated serum bone sialoprotein and osteopontin in colon, breast, prostate, and lung cancer. Clin Cancer Res.

[107] Gotoh M, Sakamoto M, Kanetaka K, Chuuma M, Hirohashi S (2002). Overexpression of osteopontin in hepatocellular carcinoma. Pathol Int.

[108] Grano M, Mori G, Minielli V, Colucci S, Vaira S, Giannelli G (2002). HGF and M-CSF modulate adhesion of MDA-231 breast cancer cell by increasing osteopontin secretion. J Biol Regul Homeost Agents.

[109] Ferrara N, Davis-Smyth T (1997). The biology of vascular endothelial growth factor. Endocr Rev.

[110] Matsui J, Funahashi Y, Uenaka T, Watanabe T, Tsuruoka A, Asada M (2008). Multi-kinase inhibitor E7080 suppresses lymph node and lung metastases of human mammary breast tumor MDA-MB-231 via inhibition of vascular endothelial growth factor-receptor (VEGF-R) 2 and VEGF-R3 kinase. Clin Cancer Res.

[111] Chien MH, Lee LM, Hsiao M, Wei LH, Chen CH, Lai TC (2013). Inhibition of Metastatic Potential in Breast Carcinoma In Vivo and In Vitro through Targeting VEGFRs and FGFRs. Evid Based Complement Alternat Med.

[112] Castanon I, Baylies MK (2002). A Twist in fate: evolutionary comparison of Twist structure and function. Gene.

[113] Furlong EE, Andersen EC, Null B, White KP, Scott MP (2001). Patterns of gene expression during Drosophila mesoderm development. Science.

[114] Yang J, Mani SA, Donaher JL, Ramaswamy S, Itzykson RA, Come C (2004). Twist, a master regulator of morphogenesis, plays an essential role in tumor metastasis. Cell.

[115] Lee TK, Poon RT, Yuen AP, Ling MT, Kwok WK, Wang XH (2006). Twist overexpression correlates with hepatocellular carcinoma metastasis through induction of epithelial-mesenchymal transition. Clin Cancer Res.

[116] Yang MH, Wu MZ, Chiou SH, Chen PM, Chang SY, Liu CJ (2008). Direct regulation of TWIST by HIF-1alpha promotes metastasis. Nat Cell Biol.

[117] Vermeulen PB, Colpaert C, Salgado R, Royers R, Hellemans H, Van Den Heuvel E (2001). Liver metastases from colorectal adenocarcinomas grow in three patterns with different angiogenesis and desmoplasia. J Pathol.

[118] Stessels F, Van den Eynden G, Van der Auwera I, Salgado R, Van den Heuvel E, Harris AL (2004). Breast adenocarcinoma liver metastases, in contrast to colorectal cancer liver metastases, display a non-angiogenic growth pattern that preserves the stroma and lacks hypoxia. Br J Cancer.

[119] Van den Eynden GG, Bird NC, Majeed AW, Van Laere S, Dirix LY, Vermeulen PB (2012). The histological growth pattern of colorectal cancer liver metastases has prognostic value. Clin Exp Metastasis.

[120] Martin MD, Kremers GJ, Short KW, Rocheleau JV, Xu L, Piston DW (2010). Rapid extravasation and establishment of breast cancer micrometastases in the liver microenvironment. Mol Cancer Res.

[121] Naumov GN, Akslen LA, Folkman J (2006). Role of angiogenesis in human tumor dormancy: animal models of the angiogenic switch. Cell Cycle.

[122] Hanahan D, Folkman J (1996). Patterns and emerging mechanisms of the angiogenic switch during tumorigenesis. Cell.

[123] Morris VL, MacDonald IC, Koop S, Schmidt EE, Chambers AF, Groom AC (1993). Early interactions of cancer cells with the microvasculature in mouse liver and muscle during hematogenous metastasis: videomicroscopic analysis. Clin Exp Metastasis.

[124] Mook OR, Van Marle J, Vreeling-Sindelarova H, Jonges R, Frederiks WM, Van Noorden CJ (2003). Visualization of early events in tumor formation of eGFP-transfected rat colon cancer cells in liver. Hepatology.

[125] Haier J, Korb T, Hotz B, Spiegel HU, Senninger N (2003). An intravital model to monitor steps of metastatic tumor cell adhesion within the hepatic microcirculation. J Gastrointest Surg.

[126] Reichen J (1999). The Role of the Sinusoidal Endothelium in Liver Function. News Physiol Sci.

[127] Roos E, Dingemans KP, Van de Pavert IV, Van den Bergh-Weerman MA (1978). Mammary-carcinoma cells in mouse liver: infiltration of liver tissue and interaction with Kupffer cells. Br J Cancer.

[128] Koo JS, Jung W, Jeong J (2010). Metastatic breast cancer shows different immunohistochemical phenotype according to metastatic site. Tumori.

[129] Wulfkuhle JD, Speer R, Pierobon M, Laird J, Espina V, Deng J (2008). Multiplexed cell signaling analysis of human breast cancer applications for personalized therapy. J Proteome Res.

[130] Jensen BV, Johansen JS, Price PA (2003). High levels of serum HER-2/neu and YKL-40 independently reflect aggressiveness of metastatic breast cancer. Clin Cancer Res.

[131] Liu J, Deng H, Jia W, Zeng Y, Rao N, Li S (2012). Comparison of ER/PR and HER2 statuses in primary and paired liver metastatic sites of breast carcinoma in patients with or without treatment. J Cancer Res Clin Oncol.

[132] Botteri E, Disalvatore D, Curigliano G, Brollo J, Bagnardi V, Viale G (2012). Biopsy of liver metastasis for women with breast cancer: impact on survival. Breast.

[133] Curigliano G, Bagnardi V, Viale G, Fumagalli L, Rotmensz N, Aurilio G (2011). Should liver metastases of breast cancer be biopsied to improve treatment choice?. Ann Oncol.

